# Risk identification of coal spontaneous combustion based on COWA modified G1 combination weighting cloud model

**DOI:** 10.1038/s41598-022-06972-4

**Published:** 2022-02-22

**Authors:** Guorui Su, Baoshan Jia, Peng Wang, Ru Zhang, Zhuo Shen

**Affiliations:** 1 College of Safety Science and Engineering, Liaoning Technical University, Fuxin, 123000 China; 2 Key Laboratory of Mine Thermodynamic Disasters and Control of Ministry of Education, Huludao, 125105 Liaoning China; 3 Ministry of Emergency Management of the People’s Republic of China, Information Institute and China Coal Information Institute, Beijing, 100029 China

**Keywords:** Energy science and technology, Mathematics and computing

## Abstract

To realize the scientific judgment of spontaneous combustion risk in the coal mine, the spontaneous combustion influence factors were analyzed from the three aspects of coal spontaneous combustion tendency, air leakage, and oxygen supply, heat storage and heat dissipation. And the basis for the evaluation of t spontaneous combustion grade was constructed. Combination ordered weighted averaging (COWA) calculation was introduced to optimizes G1 subjective weighting, and a COWA modified G1 combined weighting cloud model was proposed to identify the spontaneous combustion risk in the coal mine. Finally, the rationality of the model was verified with actual cases. The research results show that the spontaneous combustion risk level in the Lingquan coal mine is relatively safe, which is consistent with the actual situation. And the spontaneous combustion tendency of coal is the leading factor affecting spontaneous combustion risk.

## Introduction

Internal-caused fire (coal spontaneous combustion) occupies the main body of mine fire. Therefore, it is of great significance to identify the main influencing factors of internal fire and scientifically identify the risk of internal fire to ensure the intrinsic safety of the coal mine system^1,2^. Many scholars have in-depth research on the danger of spontaneous combustion in mines, and the application methods include the CW-TOPSIS model^3^, entropy weight matter-element extension theory^4,5^, gray theory^6^, set pair analysis^7–9^, chaos analysis^10^, multiple regression analysis^11^, and so on. Although the above methods have achieved certain results, there are still the following problems: first, the evaluation index weights are subject to large subjective factors. Secondly, the primary and secondary relationship between internal factors of fire is not clear. Thirdly, the extreme value of the evaluation index affects comprehensive weighting.

Based on the above considerations, the causes of spontaneous combustion are analyzed comprehensively in the mine, and a combination ordered weighted averaging (COWA) modified G1 comprehensive weighting method is proposed based on combination numbers. Combined with cloud model theory, the coal spontaneous combustion risk identification model is constructed, which provides a new method for the scientific identification of coal spontaneous combustion risk.

## Theoretical analysis of causes of fire in mine

The mine internal fire is the result of the comprehensive effect of internal and external factors of the system, in which the internal factor is the spontaneous combustion tendency of coal. And the external factor includes the air leakage and oxygen supply conditions and heat storage and heat dissipation conditions^12^. Based on the above factors, the author comprehensively analyzes the causes of mine internal fire.

### Conditions of spontaneous combustion propensity of coal

The degree of coal metamorphism reflects the physical and chemical properties of coal. The higher the degree of coal metamorphism, the higher the volatile content of coal, the easier the coal spontaneous combustion^2^. The higher the moisture content in coal, the higher the degree of coal looseness, and the higher the oxidation rate^13^. When sulfur in coal is oxidized at low temperature, the expansion of coal is loose, the oxidation area of coal is increased, and its decomposition products enhance the oxygen absorption of coal^14^. Therefore, the higher the sulfur content of coal, the easier coal spontaneous combustion. The higher the ash content in coal, the less easily broken coal, the smaller the oxidation area, the lower the possibility of spontaneous combustion^15^. Due to gas adsorption in coal, the contact between coal and oxygen is isolated, and the oxidation time of coal is prolonged^16^. Therefore, the spontaneous combustion tendency of coal is determined by five factors, including the degree of carbonization and metamorphism of coal, water content, sulfur content, ash content, and coal seam gas content.

### Air leakage oxygen supply conditions

The harder the roof, the worse the filling quality of goaf, the greater the air leakage^17^. When the thickness of residual coal is large, the contact between residual coal and oxygen increases, and the risk of spontaneous combustion of coal is higher^18^. The faster the working face advancing, the shorter the oxidation zone retention time, and the smaller the risk of spontaneous combustion^19^. Ventilation management defects affect the air leakage in goaf. When the air leakage intensity increases, the risk of coal spontaneous combustion increases^20^. Therefore, the oxygen supply conditions of air leakage are determined by four factors: roof lithology, residual coal thickness, advancing speed, and ventilation management.

### Heat storage and heat dissipation conditions

Surrounding rock temperature is an important influencing factor of heat storage in goaf. The higher the surrounding rock temperature is, the higher the risk of coal spontaneous combustion is^21^. When the depth of the coal seam is deep, the higher the initial temperature of coal is, the shorter the spontaneous combustion period of coal is^22^. The complex geological structure in coal seam affects the mining speed and prolongs the contact time between coal and air^23^. Therefore, the heat storage and dissipation conditions are determined by the surrounding rock temperature, coal seam depth, and coal seam geological structure.

### Classification of spontaneous combustion hazard

Based on the classification basis of mine spontaneous combustion^24,25^, the risk level of spontaneous combustion and influencing factors are divided into four grades: I, II, III, IV, which represent low risk, general risk , greater risk ,significant risk respectively, as shown in Table 1.

**Table 1:** Grades classification of influencing factor of coal spontaneous combustion.

Inside the mine due to fire hazards *U*_*i*_	Indicator layer	In mine due to fire danger level			
		I	II	III	IV
Spontaneous combustion of coal Tendency conditions *U*_*1*_	*U*_*11*_ Degree of charring and metamorphism of coal	0 ~ 0.2	0.2 ~ 0.5	0.5 ~ 0.8	0.8 ~ 1.0
	*U*_*12*_ Water content/%	≥ 5.5	3 ~ 5.5	1 ~ 3	≤ 1
	*U*_*13*_ Sulfur content/%	0 ~ 0.5	0.5 ~ 1.5	1.5 ~ 3.0	≥ 3.0
	*U*_*14*_ Ash content/%	≥ 24	22.5 ~ 24	21 ~ 22.5	≤ 21
	*U*_*15*_ Coal seam gas content/(m^3^/t)	≤ 4	4 ~ 10	10 ~ 16	≥ 16
Air leakage oxygen supply conditions *U*_*2*_	*U*_*21*_ Roof lithology	0 ~ 0.2	0.2 ~ 0.4	0.4 ~ 0.7	0.7 ~ 1.0
	*U*_*22*_ Thickness of relict coal/m	≤ 0.3	0.3 ~ 1.0	1.0 ~ 1.5	≥ 1.5
	*U*_*23*_ Speed of advancement/(m/d)	≥ 6	4.5 ~ 6	3 ~ 4.5	≤ 3
	*U*_*24*_ Ventilation Management	0 ~ 0.2	0.2 ~ 0.5	0.5 ~ 0.75	0.75 ~ 1.00
Heat storage and heat dissipation conditions *U*_*3*_	*U*_*31*_ Surrounding rock temperature/°C	≤ 20	20 ~ 30	30 ~ 40	≥ 40
	*U*_*32*_ Coal seam depth of burial/m	≤ 100	100 ~ 400	400 ~ 700	≥ 700
	*U*_*33*_ Geological structure	0 ~ 0.2 (no geological structure)	0.2 ~ 0.4 (simple geological structure)	0.4 ~ 0.7 (complex geological structure)	0.7 ~ 1.0 (extremely complex geological structure)

## COWA modified G1 comprehensive weighting method

### G1 subjective weight determination

The G1 method is a subjective weighting method that can reflect the importance of indicators^26^. The calculation steps are as follows:

1. Experts sorted the importance of evaluation indicators to determine the sequence relationship of indicators;
2. Determine the importance ratio of adjacent indicators *X*_*k-1*_ and *X*_*k*_;
  1 $${r}_{k}=\frac{{d}_{k-1}}{{d}_{k}}$$
  where *r*_*k*_ is the importance ratio of adjacent indicators, *d*_*k-1*_ is the importance of indicator *X*_*k-1*_, and *d*_*k*_ is the importance of indicator *X*_*k*_.
3. Based on the importance ratio *r*_*k*_ of adjacent indexes, the weight of indexes is calculated by G1 method;
  2 $${\omega }_{k}={(1+\sum_{k=2}^{n}\prod_{i=k}^{n}{r}_{i})}^{-1}$$
  3 $${\omega }_{k-1}={r}_{k}{\omega }_{k},k=n,n-1,\dots ,\mathrm{3,2}$$
  where ${\omega }_{k}$,${\omega }_{k-1}$ are the subjective weights of the *k*th, *k *− 1th indicators determined by the G1 method, and n is the total number of indicators.

### COWA operator

The COWA operator arranges the indicator data in descending order, and combines the position of the indicators for weighting, reducing the influence of subjective extreme values on indicator weights. It is an objective weighting method^27,28^. The calculation steps are as follows:

1. The index data is processed in descending order to obtain a reconstructed data set:${b}_{0}\ge {b}_{1}\ge \dots \ge {b}_{j}\ge \dots \ge {b}_{n-1}$.
2. The position weighted ${\omega }_{j+1}$ calculation of the data *b*_*j*_:
  4 $${\omega }_{j+1}=\frac{{C}_{n-1}^{j}}{{2}^{n-1}},j=\mathrm{0,1},\dots n-1$$
  where ${C}_{n-1}^{j}$ is the number of combinations of j data obtained from n-1 data.
3. Calculation of the absolute weight value of the indicator:
  5 $$\overline{{\omega  }_{i}}=\sum_{i=1}^{n}{\omega }_{j}\cdot {b}_{j}$$
  where $\overline{{\omega  }_{i}}$ is the absolute weight of the index and *b*_*j*_ is the *j*th data value.
4. Calculation of relative weight values of indicators:
  6 $${\omega }_{i}=\frac{\overline{{\omega  }_{i}}}{\sum_{i=1}^{m}\overline{{\omega  }_{i}}}$$
  where ${\omega }_{i}$ is the relative weight value of the index.

### Combination weighting under COWA correction condition

To take into account the subjectivity of decision-makers and the objectivity of data, and reduce the impact of subjective weighting extremum on weight, a COWA modified G1 combination weighting method is proposed. Based on game theory, the optimal combination of subjective weighting and objective weighting is realized by establishing combination weights and minimizing the difference between weights^29^. The calculation steps are as follows:

1. Assuming that the number of index weighting methods is m, the number of weight combinations is:
  7 $$\omega =\sum_{i=1}^{m}{u}_{i}{\omega }_{i}^{T}$$
  where *u*_*i*_ is the linear combination coefficient and ${\omega }_{i}^{T}$ is the weight of each assignment method.
2. Combinatorial coefficients ui solving.
  If the optimal point of the combinatorial assignment method is realized, the optimization model can be constructed as:
  8 $${min\Vert \sum_{j=1}^{m}{u}_{j}{\omega }_{j}^{T}-{\omega }_{i}^{T}\Vert }_{2}$$
  Then the first-order derivative condition for its optimal condition is:
  9 $$\sum_{j=1}^{m}{u}_{j}{\omega }_{i}{\omega }_{j}^{T}={\omega }_{i}{\omega }_{i}^{T}$$
3. Combined coefficients ui normalization processing:
  10 $${u}_{i}^{*}=\frac{{u}_{i}}{\sum_{i=1}^{m}{u}_{i}}$$
4. Based on the above analysis, the optimal combination weights are:
  11 $${\omega }^{*}=\sum_{i=1}^{m}{u}_{i}^{*}{\omega }_{i}^{T}$$

## Model for identifying the risk of spontaneous combustion in mines

### Cloud model theory

Cloud models enable the uncertain transformation of qualitative concepts and quantitative descriptions by establishing a mapping relationship between quantitative and qualitative concepts^30,31^.

1. Cloud model definition and its numerical characteristics
  In mine endogenous fire evaluation, assuming that *U* is the theoretical domain corresponding to the values of endogenous fire indicators, *C* is the qualitative concept in endogenous fire evaluation indicators, *x* denotes cloud drops, and *u*(*x*) is the affiliation degree of any cloud drop *x* to *C* in the theoretical domain *U*, then12
  The cloud model is represented by expectation (*Ex*), entropy (*En*), and superb entropy (*He*). *Ex* is the center of the cloud graph, *En* characterizes the reliability of *Ex*, and *He* characterizes the uncertainty of *En*. The distribution interval of cloud drops *x* is [*Ex*-*3En*, *Ex* + *3En*].
2. Cloud Generator

The cloud generator is divided into forward and inverse cloud generators. The forwarding cloud generator mainly realizes the conversion from qualitative concept to quantitative, and the calculation steps are as follows:

① With expectation *En*, variance *He*^*2*^, generate Gaussian random number ${E}_{{n}_{i}}^{^{\prime}}=NORM(En,{He}^{2})$.
② With the expected value of Ex, variance ${{E}_{{n}_{i}}^{^{\prime}}}^{2}$ Constructing Gaussian random numbers ${x}_{i}=NORM(En,{{E}_{{n}_{i}}^{^{\prime}}}^{2})$.
③ Calculation of the determination of the indicator:
  13 $${\psi }_{i}=\mathrm{exp}[-\frac{{({x}_{i}-Ex)}^{2}}{2{\left(En\right)}^{2}}]$$
  where ${\psi }_{i}$ is the degree of determination of the index.
④ Cloud droplet interval construction, based on the above steps to form a cloud droplet (*x*_*i*_,*u*_*i*_), and then repeat steps ① ~ ③, until the formation of *N* cloud droplets.

### Cloud numerical characteristics of fire hazard indicators in mines

Referring to the related research results^32^, the cloud number characteristics of the fire hazard index in the mine can be calculated as follows,

14 $$\left\{\begin{array}{c}Ex=\left({F}_{min}+{F}_{max}\right)/2\\ En=\left({F}_{max}{-F}_{min}\right)/6\\ He=k\end{array}\right.$$

where *F*_*max*_ is the upper limit of the index value, *F*_*min*_ is the lower limit of the index value, and *k* is the degree of index fuzziness, which is taken as 0.05 here.

Then, based on the graded values of Table 1 indicators, the cloud numerical characteristics of the mine endogenous fire evaluation indicators are calculated by Eq. (14), and for variables with unilateral boundaries, their numerical characteristics are obtained in the form of boundary parameters^33,34^, as shown in Table 2.

**Table 2:** Numerical characteristics of the cloud model for an index of coal spontaneous combustion.

Indicators	Grade I (*Ex, En, He*)	Grade II (*Ex, En, He*)	Grade III (*Ex, En, He*)	Grade IV (*Ex, En, He*)
*U*_*11*_	(0.10, 0.03, 0.05)	(0.35, 0.05, 0.05)	(0.65, 0.05, 0.05)	(0.90, 0.03, 0.05)
*U*_*12*_	(5.50, 0.42, 0.05)	(4.25, 0.42, 0.05)	(2.00, 0.33, 0.05)	(0.50, 0.17, 0.05)
*U*_*13*_	(0.25, 0.08, 0.05)	(1.00, 0.17, 0.05)	(2.25, 0.25, 0.05)	(3.00, 0.25, 0.05)
*U*_*14*_	(24.00, 0.25, 0.05)	(23.25, 0.25, 0.05)	(21.75, 0.25, 0.05)	(10.50, 3.50, 0.05)
*U*_*15*_	(2.00, 0.67, 0.05)	(7.00, 1.00, 0.05)	(13.00, 1.00, 0.05)	(16.00, 1.00, 0.05)
*U*_*21*_	(0.10, 0.03, 0.05)	(0.30, 0.03, 0.05)	(0.55, 0.05, 0.05)	(0.85, 0.05, 0.05)
*U*_*22*_	(0.15, 0.05, 0.05)	(0.65, 0.12, 0.05)	(1.25, 0.08, 0.05)	(1.50, 0.08, 0.05)
*U*_*23*_	(6.00, 0.25, 0.05)	(5.25, 0.25, 0.05)	(3.75, 0.25, 0.05)	(1.50, 0.50, 0.05)
*U*_*24*_	(0.10, 0.03, 0.05)	(0.35, 0.05, 0.05)	(0.625, 0.04, 0.05)	(0.875, 0.04, 0.05)
*U*_*31*_	(10.00, 3.33, 0.05)	(25.00, 1.67, 0.05)	(35.00, 1.67, 0.05)	(40.00, 1.67, 0.05)
*U*_*32*_	(150.00, 16.70, 0.05)	(300.00, 33.30, 0.05)	(550.00, 50.00, 0.05)	(700.00, 50.00, 0.05)
*U*_*33*_	(0.10, 0.03, 0.05)	(0.30, 0.03, 0.05)	(0.55, 0.05, 0.05)	(0.85, 0.05, 0.05)

### Comprehensive discriminative model construction

Based on the cloud numerical characteristics of the evaluation indexes of mine internal fires, Matlab software is used to generate the cloud diagram of evaluation indexes, determine the determinacy of each index under different hazard levels, and then combine the G1 combination weights under COWA correction conditions to obtain the comprehensive rating of mine internal fires.

15 $$U=\sum_{j=1}^{n}{\psi }_{x}{\omega }^{*}$$

where ${\psi }_{x}$ is the single indicator determinant and ${\omega }^{*}$ is the optimal combination weight.

## Case analysis

### Determination of index weights

The II 3 coal seam of Lingquan Coal Mine in Inner Mongolia is selected as an application example, the degree of charring and metamorphism of coal is 0.32, the coal seam buried depth 447 ~ 470 m; coal seam gas content 7.8m^3^/t; the geological structure is simple, the average thickness of coal remains 1.2 m, the average speed of advance 9 m/d, the average thickness 14.69 m, the lithology of the roof is medium sandstone, the measured water content 3.19%, ash content 12.14%, sulfur content 0.16%, the temperature of the surrounding rock is about 23 °C, and the natural firing period is 40d, which is easy to the spontaneous combustion coal seam. The values of qualitative and quantitative indicators were determined by expert experience, and based on the principle of weight determination by the G1 method, industry experts were hired to analyze the serial relationship and importance among the indicators, and calculate the subjective weights according to Eqs. (1)–(3), while determining the objective weights based on COWA operator according to Eqs. (4)–(6), and finally combining Eqs. (7)–(11) to determine the comprehensive weights of the indicators, as shown in Table 3.

**Table 3:** Index weight and cloud numerical characteristics distribution of coal spontaneous combustion.

Secondary indicator	Three-level indicators	G1 method		COWA method		Combination weighting method	
		Secondary level	Third level	Secondary level	Third level	Secondary level	Third level
U_1_	U_11_	0.473	0.075	0.456	0.066	0.462	0.070
	U_12_		0.112		0.124		0.118
	U_13_		0.081		0.091		0.086
	U_14_		0.101		0.112		0.106
	U_15_		0.092		0.108		0.098
U_2_	U_21_	0.298	0.041	0.309	0.027	0.305	0.037
	U_22_		0.052		0.040		0.047
	U_23_		0.068		0.058		0.063
	U_24_		0.038		0.021		0.029
U_3_	U_31_	0.229	0.134	0.235	0.146	0.233	0.139
	U_32_		0.139		0.151		0.146
	U_33_		0.067		0.056		0.061

### Determination and analysis of risk grade

Based on the cloud numerical characteristics of mine endogenous fire indicators in Table 2, the risk level determinacy of each indicator was calculated by Eq. (13), and then combined with the comprehensive weights of each indicator of endogenous fire in Table 3, the comprehensive determinacy of the hazard evaluation model was calculated by Eq. (15) to determine the hazard level of endogenous fire in Lingquan coal mine. The determinacy of the risk level of endogenous fire in Lingquan coal mine was calculated as P(I) = 0.046, P(II) = 0.260, P(III) = 0.074, P(IV) = 0.095, respectively, and it is known that the risk boundary of endogenous fire in Lingquan coal mine is level II according to the principle of maximum determinacy, which is consistent with the actual situation of the mine. The calculation method and evaluation criterion of reference^3^ were selected to analyze the risk level of endogenous fire in the Lingquan coal mine, and the result was a general risk level, and the results of the two methods were consistent, which showed the reasonableness of the COWA modified G1 combined assignment cloud model in the evaluation of endogenous fire in the mine.

Meanwhile, the weights of each index in Table 3 were analyzed, and the weights were arranged in descending order, in which the secondary index sequence was spontaneous combustion propensity condition of coal > air leakage and oxygen supply condition > heat storage and heat dissipation condition, and the tertiary main index sequence was coal seam depth > surrounding rock temperature > water content > ash content > sulfur content, and the results were approximately the same as the findings of references^3^, which verified that the COWA modified G1 combined assignment weighting method was more effective in determining the endogenous fire risk. The scientific feasibility of the COWA modified G1 combination weighting method in determining the weights of fire indicators.

## Conclusion

1. Considering the three aspects of coal spontaneous combustion tendency condition, air leakage oxygen supply, and heat storage, the grade evaluation basis of fire risk index in mine is constructed.
2. The COWA modified G1 combination weighting cloud model is proposed to identify the fire risk in the mine, and the Lingquan coal mine is taken as the engineering background for verification. The risk level is relatively safe, which is consistent with the actual scene, and is consistent with the conclusion of the CW-TOPSIS method.
3. Based on the COWA modified G1 combination assignment method, the weights of each index are analyzed in descending order, among which, among the secondary indexes, the propensity of coal to spontaneous combustion has the greatest influence on mine internal fire, and among the tertiary indexes, the depth of coal seam, the temperature of surrounding rock, water content and ash content are the main factors affecting mine internal fire. Based on the COWA modified G1 combination assignment method, the weights of each index are analyzed in descending order, among which, among the secondary indexes, the propensity of coal to spontaneous combustion has the greatest influence on mine internal fire, and among the tertiary indexes, the depth of coal seam, the temperature of surrounding rock, water content and ash content are the main factors affecting mine spontaneous combustion.
